# Uncovering the genetic diversity of yams (*Dioscorea* spp.) in China by combining phenotypic trait and molecular marker analyses

**DOI:** 10.1002/ece3.7727

**Published:** 2021-07-16

**Authors:** Tianxu Cao, Jingyu Sun, Nan Shan, Xin Chen, Putao Wang, Qianglong Zhu, Yao Xiao, Hongyu Zhang, Qinghong Zhou, Yingjin Huang

**Affiliations:** ^1^ Agronomy College Jiangxi Agricultural University Nanchang China; ^2^ School of Advanced Agriculture and Bioengineering Yangtze Normal University Chongqing China; ^3^ Key Laboratory of Crop Physiology, Ecology and Genetic Breeding Jiangxi Agricultural University Ministry of Education of China Nanchang China

**Keywords:** genetic diversity, molecular markers, phenotypic trait, population structure, yams

## Abstract

Yam is an important edible tuber and root plant worldwide; China as one of the native places of yams has many diverse local resources. The goal of this study was to clarify the genetic diversity of the commonly cultivated yam landraces and the genetic relationship between the main yam species in China. In this study, 26 phenotypic traits of 112 yam accessions from 21 provinces in China were evaluated, and 24 simple sequence repeat (SSR) and 29 sequence‐related amplified polymorphism (SRAP) markers were used for the genetic diversity analysis. Phenotypic traits revealed that *Dioscorea opposita* had the highest genetic diversity, followed by *D. alata*, *D. persimilis*, *D. fordii*, and *D. esculenta*. Among the 26 phenotypic traits, the Shannon diversity indexes of leaf shape, petiole color, and stem color were high, and the range in the variation of tuber‐related traits in the underground part was higher than that in the aboveground part. All accessions were divided into six groups by phenotypic trait clustering, which was also supported by principal component analysis (PCA). Molecular marker analysis showed that SSR and SRAP markers had good amplification effects and could effectively and accurately evaluate the genetic variation of yam. The unweighted pair‐group method with arithmetic means analysis based on SSR‐SRAP marker data showed that the 112 accessions were also divided into six groups, similar to the phenotypic trait results. The results of PCA and population structure analysis based on SSR‐SRAP data also produced similar results. In addition, the analysis of the origin and genetic relationship of yam indicated that the species *D. opposita* may have originated from China. These results demonstrate the genetic diversity and distinctness among the widely cultivated species of Chinese yam and provide a theoretical reference for the classification, breeding, germplasm innovation, utilization, and variety protection of Chinese yam resources.

## Graphical abstract

Representative yam resources from five species were collected in 21 provinces in China. The genetic diversity of 112 yam accessions in China was evaluated roundly by combining phenotypic traits, SSR, and SRAP markers. Genetic difference between yam resources in China was identified roughly, and *Dioscorea* *opposita* probably originated from China.

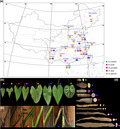

## 1 INTRODUCTION

Yam is the common name for over 600 *Dioscorea* species. The species of yams widely cultivated worldwide are *D. bulbifera*, *D. panthaica*, *D. esculenta*, *D. japonica*, *D. trifida*, *D. pentaphylla,* and *D. rotundata* (Lebot, 2009). Yams are also one of the top 10 most important edible tuber and root plants worldwide and are next to potato, cassava, and sweet potato in yield (Shewry, 2003). Yams play an important role in sustaining many livelihoods in the tropics and subtropics (Tschannen et al., 2005; Wu et al., 2019).

Yams likely originated from cultivation and domestication centers in Asia, Africa, and America over long‐term evolution. The African group, including the species *D. rotundata*, *D. cayennensis,* and *D. dumetorum,* has become the main regional belt of yam production worldwide; these yams are mainly distributed in Ghana, Togo, Benin, and other West African regions, Central Africa, and the Western Congo (Coursey, 1976; Martin & Sadik, 1977). *D. trifida* is the earliest known domesticated variety in South America and is widely cultivated, mainly in Brazil, Venezuela, Paraguay, and other regions (Coursey, 1976). Asia is another main distribution center of yam, and the commonly cultivated and domesticated species in Asia are *D. opposita,* *D. alata,* *D. esculenta,* *D. japonica,* *D. bulbifera,* *D. hispida,* and *D. quinquelaba* (Gong et al., 2004).

China is one of the important domestication centers of yams such as *D. opposita*, *D. alata*, and the records of *D. opposita* dating back to “the Classic of Mountains and Rivers” more than 4,000 years ago (Yuan, 1980). In China, yam is known as a medicinal and edible crop with high nutritional and medicinal value; its tuber is rich in starch, protein, and medicinal ingredients (e.g., allinogenin, diosgenin, and dehydroepiandrosterone; Lebot et al., 2019). The cultivar “Tiegun” is one of the most popular *D. opposita* cultivars and has been used for more than 2000 years to treat conditions such as diarrhea, diabetes, and asthma (Peng et al., 2017). In China, yam resources are extremely rich, with a total of 65 species (Guo & Liu, 1994). *D. opposita*, *D. alata*, *D. persimilis*, *D. fordii*, and *D. quinquelaba* are widely cultivated (Huang et al., 2011). Yams are cultivated in all provinces, except Qinghai and Tibet, and include a large number of landraces. However, yams have long been regarded as “orphan” or “neglected” crops despite their considerable edible and medicinal value and have received little attention or investment from researchers (Tamiru et al., 2017). Moreover, *Dioscorea* is mainly dioecious, rarely flowers, and has difficulty forming mature seeds (Bressan et al., 2011). The development of medicinal ingredients from a few species of yam has long been emphasized in China (Cheng et al., 2020; Lebot et al., 2018; Li et al., 2018), but the analysis of the resource types and genetic diversity of yam is insufficient. In addition, most studies on the genetic diversity of yam are focused on *D. alata* (Arnau et al., 2017; Siqueira et al., 2014; Wu et al., 2019), while research on *D. opposita*, as the most popular species with the largest cultivated area in China, is rarely reported. In the long‐term cultivation and domestication process, the varieties of yam are complex, and a single classification method has been difficult to identify, thereby causing confusion across various records and nomenclatures and even in the classification of some species. These factors seriously hinder resource conservation and the further utilization of yam.

Therefore, studying the genetic diversity, genetic variation, and population structure of yam is highly important to its origin, distribution, resource utilization, parental selection, and development (Mignouna et al., 2003). To date, phenotypic traits, karyotype analysis, and DNA diversity have been used to describe the genetic diversity of yam germplasm (Cao et al., 2020; Kouam et al., 2018; Nemorin et al., 2013; Sartie et al., 2012). Phenotypic traits are important for the identification and effective utilization of germplasm resources (Daley et al., 2020). Although morphological traits are easy to measure, they are subject to many limitations and are particularly dependent on the environment. However, molecular markers are not affected by environmental factors and have been effectively applied in plant systematics, breeding, and gene resource assessment (Naval et al., 2010). Simple sequence repeat (SSR) markers are widely used markers in the fields of ecology, biology, and genetics, with the advantages of codominance, high occurrence in genomes, and high polymorphism (Chapman et al., 2009). Sequence‐related amplified polymorphism (SRAP) PCR markers target the open reading frame (ORF) and combine simplicity, reliability, moderate throughput, and convenient band sequencing. In addition, SRAP targets coding sequences in the genome and generates a moderate number of codominant markers (Li & Quiros, 2001). SSR and SRAP markers have a high degree of polymorphism, which is useful for the identification of germplasm resources, and the application of these two markers has effectively produced a large amount of reliable genetic data (Dong et al., 2019; György et al., 2016). The methods described above have been widely used in the identification of genetic diversity and genetic relationships of yam germplasm resources (Anokye et al., 2014; Mignouna, Dansi, et al., 2002; Mignouna, Mank, et al., 2002; Silva et al., 2017). At present, there are some reports on the genetic diversity of *D. alata* in China (Wu et al., 2009, 2019). However, there are a few reports on the genetic variation and structure of Chinese yam based on the combination of molecular and morphological markers for a wide range of germplasm locations.

The goal of this study was to clarify the genetic diversity of the commonly cultivated yam landraces and the genetic relationship between the main yam species in China. In the current study, 106 yam landraces and 6 wild resources from 5 species widely used for cultivation were collected in 21 provinces, and their genetic diversity, genetic relationship, population structure, and interspecific genetic relationship were comprehensively identified and evaluated by combining phenotypic traits with SRAP and SSR molecular markers. This study will provide the basis for the identification, classification, and breeding of Chinese yam landraces and provide a theoretical reference for the exploration of the origin and domestication of yam.

## 2 MATERIALS AND METHODS

### 2.1 Plant materials

**FIGURE 1:**
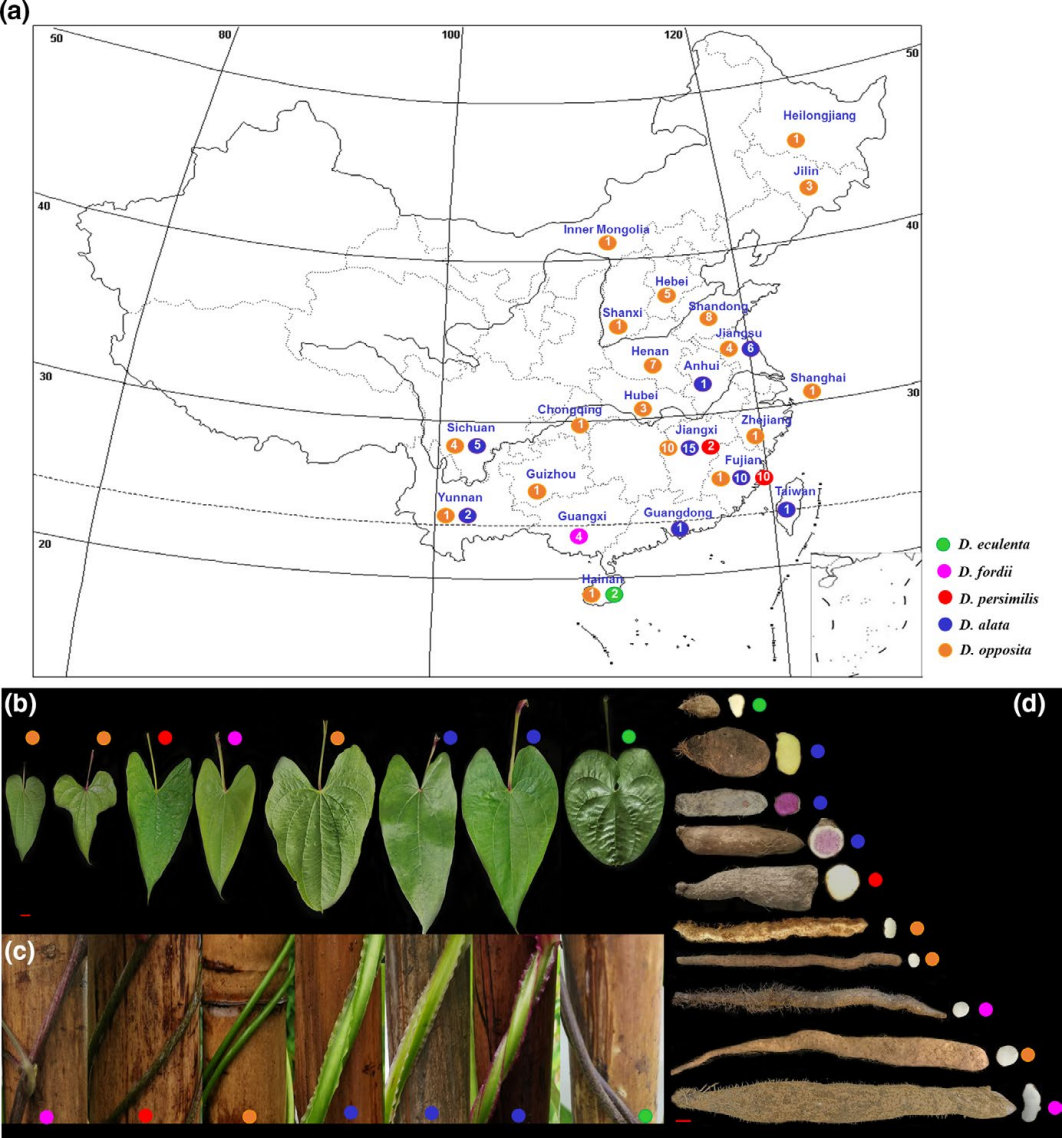
The geographical distribution of the different yam species and the number of yam accessions in different provinces (a) and images of leaves (b), stems (c), and tubers (d) of five yam species in China. The number indicates the quantity of all resources collected in each province, and the solid circles with different colors indicate different yam species. Bar = 1 cm

A total of 112 yam accessions widely cultivated were collected from 21 provinces in China, including the species *D. opposita* (53), *D. alata* (41), *D. persimilis* (12), *D. fordii* (4), and *D. esculenta* (2), which are currently the main cultivated species in China (Figure 1a and Table S1). Landraces (106) were collected from farmers’ fields, institutions, and markets in China, and wild resources (6) were acquired from mountainous regions. All accessions were planted in the yam germplasm resource garden of Jiangxi Agricultural University (Nanchang City, Jiangxi Province). Experimental planting was arranged in ridges on 10 April 2019, with a 20 cm distance between each individual plant and a 1.2 m distance between ridges. Tuber segments (80–120 g) were used as “propagules”. Individual plants were supported by bamboo stakes. Standard weeding and agronomic measures were applied regularly to provide adequate plant growth conditions. Three replicates were performed for each accession, and 10 individual plants were planted in each replicate.

### 2.2 Phenotype assessment

A total of 26 phenotypic traits of leaves, stems, flowers, aerial stems, tubers, and roots of yam were evaluated (Table 1), including 20 qualitative traits and 6 quantitative traits. The aboveground phenotypic traits were investigated 60–90 days after planting, the stem and leaf‐related traits were investigated about 60 days after planting, and the traits such as flowers and aerial tubers were investigated about 90 days after planting. The investigation of the traits related to underground tubers was conducted in October and November after harvest. Phenotypic traits were observed in the field, and data recording was performed as previously described (Huang & Huang, 2013; IPGRI/IITA, 1997; Wang & Shen, 2014). Six individual plants were randomly selected for each accession to observe the qualitative traits and the measured values of the quantitative traits.

**TABLE 1:** Descriptors used for the phenotypic assessment of yam accessions in this study

	Traits	Classes (Codes)
1	Flowering (FL)	1 = Absent, 2 = Male, 3 = Female.
2	Aerial tubers (AT)	1 = Present, 2 = Absent.
3	Leaf shape (LS)	1 = Heart triangle, 2 = Triangular ovate, 3 = Lanceolate, 4 = Round, 5 = Shape of halberd.
4	Leaf color (LC)	1 = Yellow‐green, 2 = Greenish gray, 3 = Dark green.
5	Leaf apex shape (LAX)	1 = Obtuse, 2 = Acute.
6	Distance between lobes (DBL)	1 = Intermediate, 2 = Very distant, 3 = No measurable distance.
7	Leaf margin color (LMC)	1 = Green, 2 = Purple.
8	Petiole color (PC)	1 = Purple, 2 = Green, 3 = Greenish purple, 4 = Purplish‐red.
9	Leaf vein color (LVC)	1 = Yellow‐green, 2 = Green, 3 = Purple.
10	Leaf vein (LV)	1 = Five, 2 = Seven veins, 3 = Nine veins.
11	Stem wing (SW)	1 = Absent, 2 = Present.
12	Stem color (SC)	1 = Green, 2 = Green with purple, 3 = Brownish green, 4 = Purple.
13	Stem spine (SSP)	1 = Absent, 2 = Present.
14	Twining direction (TD)	1 = Anticlockwise, 2 = Clockwise.
15	Tuber shape (TS)	1 = Oval, 2 = Cylindrical, 3 = Irregular.
16	Roots hair density (RHD)	1 = Sparse, 2= Dense.
17	Place of roots on the tuber (PRT)	1 = All, 2 = Upper and Middle
18	Tuber skin color (TSC)	1 = Brown, 2 = Black, 3 = Gray.
19	Tuber skin color under bark (TSCUB)	1 = Beige, 2 = Purple.
20	Flesh color (FC)	1 = White, 2 = Yellow, 3 = Purple, 4 = Purple with white.
21	Leaf length (LL)	Average leaf length of six mature leaves (cm).
22	Leaf width (LW)	Average leaf width of six mature leaves (cm).
23	Length‐to‐width ratio (L/W)	Average leaf length/average leaf width.
24	Tuber length (TL)	Average tuber length of six plants (cm).
25	Tuber diameter (TD)	Average tuber diameter of six plants (mm).
26	Tuber flesh weight (TFW)	Average yield of six plants (g).

### 2.3 DNA extraction

DNA samples of the accessions were isolated from young leaves by using a plant genomic DNA kit (TaKaRa MiniBEST plant genomic DNA Extraction Kit, TaKaRa, Beijing, China). The DNA samples were quantified using a NanoDrop 2000 (Wilmington, USA) spectrophotometer and checked on 1.0% (w/v) agarose gels stained with ethidium bromide. Based on concentration estimations, all samples were diluted to 20 ng/μl and stored at −20°C.

### 2.4 SSR and SRAP genotyping

Twenty‐four SSR markers (Table S2) with polymorphic bands in all accessions were selected for further analysis from the initial 53 SSR markers that produced amplicons (Loko et al., 2016; Narina et al., 2011; Nemorin et al., 2013). The primers were synthesized by Sangon Biotech (Shanghai) Co., Ltd. PCR amplification reactions were performed using a master mix solution of 10 μl containing 5 μl of 2 × Master Mix Blue (TSINGKE, China), 0.25 μl of each primer (10 mM), and 0.75 μl of template DNA (20 ng/μl), and the remaining volume was supplemented with ddH_2_O. The following cycling parameters were used in the amplification reaction: first predenaturation at 94°C for 5 min, followed by 40 cycles of 30 s at 94°C, annealing for 30 s at 54°C, and 30 s at 72°C, and a final extension of 10 min at 72°C. The amplified PCR products were detected on an 8% nondenaturing polyacrylamide gel. Silver nitrate staining was employed, and images were captured for analysis.

Forty‐nine different SRAP primers were obtained from the combination of seven forward primers and seven reverse primers (Li & Quiros, 2001; Table S3), of which 29 primer combinations with good repeatability and high polymorphism were selected for this study. Each 14 μl PCR mixture consisted of 7 μl of 2 × Master Mix Blue, 0.35 μl of each primer (10 mM), and 1.4 μl of template DNA (20 ng/μl), and the remaining volume was supplemented with ddH_2_O. PCR amplification was performed under the following conditions: denaturation at 94°C for 5 min, five cycles of three steps: denaturation at 94°C for 1 min, annealing at 35°C for 1 min, and elongation at 72°C for 1 min. In the following 30 cycles, the annealing temperature was increased to 56°C, with a final extension step of 10 min at 72°C. The amplified products were analyzed through 3% agarose gel electrophoresis prepared in 1× TBE buffer. The gels were then visualized in a UV transilluminator (Bio‐Rad GeL Doc XR+, USA) and photodocumented.

### 2.5 Data analysis

The survey results of 20 qualitative traits were classified and assigned different values according to Table 1. The distribution frequency of each classification was also calculated. Then, the Shannon diversity index (*I*) was calculated in accordance with the distribution frequency as follows:

$$I=\sum_{i=1}^{n}\left(pi)(\ln\;pi\right),$$

where *p_i_* represents the relative frequency of the *i*th phenotypic class of a trait (Kouam et al., 2018).

The maximum, minimum, average, standard deviation (*SD*), and coefficient of variation (CV) of six quantitative traits were calculated using SPSS 25.0 software. Then, in accordance with the overall average ($\bar{x}$) and *SD* (*σ*), the quantitative trait data were divided into 10 levels, from the first level $\left[Xi<(\bar{x}‐2\sigma )\right]$ to the 10th level $\left[Xi>(\bar{x}+2\sigma )\right]$, in increments of 0.5 *σ*. In accordance with the phenotypic trait survey data, a matrix (1, 0) was constructed, and the registration at the *i*th level of a trait was 1; otherwise, it was 0.

The polymorphic bands of SRAP and SSR markers were labeled as present (1) and absent (0) for each primer at each locus, and the binary matrix was constructed and statistically analyzed. The observed number of alleles (*N*_a_), effective number of alleles (*N*_e_), allele frequency, Shannon's diversity index (*I*), observed heterozygosity (H_o_), expected heterozygosity (*H*_e_), and Nei's (1973) gene diversity index (*H*) of each SSR and SRAP primer were calculated with POPGENE software version 1.32 (Yeh Francis et al., 1999). Principal component analysis (PCA) of phenotypic traits and molecular markers uses OmicShare, a free online platform for data analysis (www.omicshare.com/tools). Cluster analysis of the unweighted pair‐group method with arithmetic means of the phenotypic traits and molecular markers was performed using MEGA software version 4.1 (Tamura et al., 2007). Based on the combined data of SRAP and SSR, the population structure of all accessions, *D. opposita* separately, and *D. alata* separately, was analyzed by Bayesian model in STRUCTURE software version 2.3.1 (Pritchard et al., 2000). *K* (number of clusters) was estimated to be in the range of 2–10, and the software was run ten times to determine this value. Estimates were obtained with the Markov chain Monte Carlo (MCMC) method with 100,000 iterations followed by a burn‐in period of 500,000 iterations. STRUCTURE HARVESTER (Earl & Vonholdt, 2012), which determines the best K based on the probability of data given K and ΔK (Evanno et al., 2005), was used to estimate the most likely number of clusters (K).

## 3 RESULTS

### 3.1 Phenotypic diversity analysis

#### 3.1.1 Analysis of qualitative and quantitative traits

**FIGURE 2:**
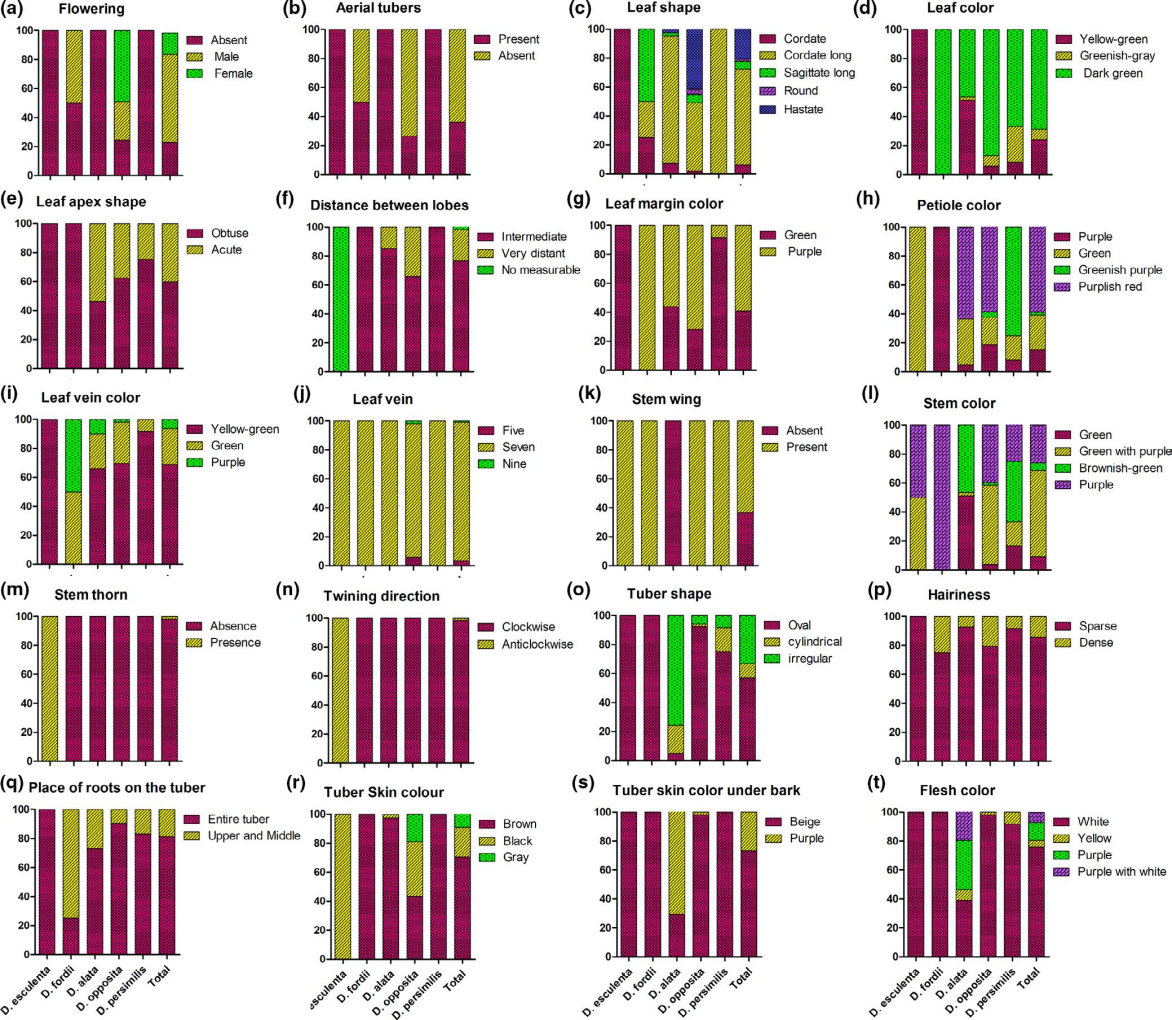
The distribution frequency (%) of each qualitative trait in five yam species. The sample sizes of *D. opposita*, *D. alata*, *D. persimilis*, *D. fordii,* and *D. esculenta* are 53, 41, 12, 4, and 2, respectively

Twenty qualitative traits showed great variability across all accessions, and the *I* values ranged from 0.09 to 1.03, with an average value of 0.650 (Table 2). For the five species, the *I* value of *D. opposita* was the highest, followed by those of *D. alata*, *D. persimilis*, *D. fordii,* and *D. esculenta* (Table 2). The *I* values for leaf shape, petiole color, and stem color were greater than 1 (Table 2). The trait with the highest diversity was stem color (*I* = 1.030), while the *I* values of stem thorn and twining direction were the lowest (0.090, Table 2). Similar results could be obtained from the distribution frequency (%) of each trait in the five species (Figure 2). For leaf shape, *D. esculenta* and *D. persimilis* had no variation and were round and triangular ovate, respectively (Figure 1b). However, the other three species all had variation in leaf shape, especially *D. opposita,* which had the largest variation (Figure 2c). The petiole color of all accessions of *D. fordii* was purple, while the petiole color of all accessions of *D. esculenta* was green, and the petioles of the other species were purple, green, greenish purple, and purplish‐red (Figure 2h). For stem color, only the accessions of *D. fordii* were purple without variation, while the other species showed variation (Figures 1c and 2l). Twining direction, the distance between lobes and stem thorn for *D. esculenta,* showed no diversity, while the other species had diversity in these traits (Figure 2f,m,n). Only the accessions of *D. alata* had stem wings, accounting for 36.61% of all accessions; this trait could be used as a phenotypic marker to identify this species. (Figures 1c and 2k). No flowering was found in accessions of *D. esculenta*, *D. alata,* and *D. persimilis*. Fifty percent of the accessions of *D. fordii* flowered, and all flowers were male, while 75% of the accessions of *D. opposita* flowered and 50% were female (Figure 2a). Aerial tubers were found in 50% of the accessions of *D. fordii* and 73.58% of the accessions of *D. opposita*, while no aerial tubers were observed in the other three species in the current study (Figure 2b). Here, other qualitative traits also showed different levels of diversity across the accessions of the five species (Figure 2).

**TABLE 2:** The Shannon diversity index (I) of 20 qualitative traits in five yam species

Traits	Diversity index (*I*)					
	*D. esculenta*	*D. fordii*	*D. alata*	*D. opposita*	*D. persimilis*	Total
FL	0	0.693	0	1.043	0	0.924
AT	0	0.693	0	0.557	0	0.662
LS	0	1.040	0.487	1.081	0	1.001
LC	0	0	0.790	0.481	0.824	0.789
LAX	0	0	0.691	0.663	0.562	0.674
DBL	0	0	0.416	0.641	0	0.605
LMC	0	0	0.686	0.596	0.287	0.677
PC	0	0	0.800	1.067	0.722	1.013
LVC	0	0.693	0.846	0.683	0.287	0.778
LV	0	0	0	0.310	0	0.174
SW	0	0	0	0	0	0.657
SC	0.693	0	0.416	0.895	1.309	1.030
SSP	0	0	0	0	0	0.090
TD	0	0	0	0	0	0.090
TS	0	0	0.678	0.310	0.722	0.914
RHD	0	0.562	0.262	0.511	0.287	0.410
PRT	0	0.562	0.582	0.313	0.451	0.483
TSC	0	0	0.115	1.045	0	0.787
TSCUB	0	0	0.605	0.094	0	0.581
FC	0	0	1.244	0.094	0.287	0.797
Mean	0.035	0.212	0.431	0.519	0.287	0.657

For all accessions, the CVs of the six quantitative traits ranged from 20.81% to 76.11%, and the CVs of tuber fresh weight, tuber length, tuber diameter, and leaf length were all more than 30%, with the highest values for tuber fresh weight and the lowest for length‐to‐width ratio. The range of tuber fresh weight values changed the most (1643.73 g), followed by tuber length (89.92 cm), tuber diameter (15.75 mm), leaf length (13.39 cm), and leaf width (8.88 cm; Table 3). Among the five species, the CV of leaf length in *D. fordii* was the highest (23.97%), and that in *D. esculenta* was the lowest (4.39%). The CV of leaf width in *D. opposita* was the highest (21.17%) and that of *D. persimilis* was the lowest (3.55%). The CV of the ratio of length‐to‐width of leaves was the highest in *D. alata* and the lowest in *D. esculenta*. *D. persimilis* had the highest CV of tuber length, and the highest CVs of tuber diameter and tuber fresh weight were both found in *D. opposita*. The CVs of tuber length, tuber diameter, and tuber fresh weight in *D. esculenta* were the lowest (Table 3). Table 3 shows that the range of variation in tuber‐related traits in the underground part was larger than that in the aboveground part in each species, and *D. opposita*, *D. alata*, *D. persimilis,* and *D. fordii* showed higher variation in quantitative traits than *D. esculenta*. Phenotypic analysis showed that the commonly cultivated species of Chinese yam had rich diversity, especially *D. opposita*, *D. alata*, *D. persimilis,* and *D. fordii*. However, the diversity of *D. esculenta* was low, and the number of accessions might be one of the important reasons; further analysis will require a large number of accessions.

**TABLE 3:** Statistical analysis of intraspecific and interspecific quantitative traits of five yam species

Species	Trait	Mean	Maximum	Minimum	Range	*SD*	CV (%)
*D. esculenta*	LL (cm)	8.90	9.47	0.57	9.19	0.40	4.39
	LW (cm)	8.70	9.80	1.10	9.25	0.78	8.41
	L/W	0.97	1.02	0.05	1.00	0.04	3.55
	TL (cm)	7.50	7.80	0.30	7.65	0.21	2.77
	TD (mm)	4.80	5.30	0.50	5.05	0.35	7.00
	TFW (g)	78.60	107.00	28.40	92.80	20.08	21.64
*D. fordii*	LL (cm)	8.50	14.80	6.30	12.38	2.97	23.97
	LW (cm)	4.82	6.70	1.88	5.77	0.95	16.45
	L/W	1.76	2.29	0.53	2.13	0.25	11.75
	TL (cm)	49.60	66.50	16.90	57.78	6.94	12.02
	TD (mm)	7.00	10.10	3.10	8.65	1.29	14.88
	TFW (g)	621.00	1,108.50	487.50	847.23	202.25	23.87
*D. alata*	LL (cm)	10.54	19.27	8.73	14.92	2.31	15.49
	LW (cm)	4.85	11.73	6.88	8.29	1.63	19.69
	L/W	0.95	3.25	2.30	1.84	0.33	17.96
	TL (cm)	7.25	62.70	55.45	22.87	11.02	48.17
	TD (mm)	5.10	17.50	12.40	10.78	3.42	31.73
	TFW (g)	5.60	1,649.33	1,643.73	726.29	445.48	61.34
*D. opposita*	LL (cm)	5.88	16.00	10.12	8.62	1.73	20.11
	LW (cm)	2.85	9.61	6.76	6.02	1.31	21.71
	L/W	1.10	2.20	1.10	1.46	0.23	15.85
	TL (cm)	17.50	77.50	60.00	40.46	12.58	31.10
	TD (mm)	1.75	15.38	13.63	6.65	2.95	44.36
	TFW (g)	61.50	1,316.00	1,254.50	350.72	250.60	71.45
*D. persimilis*	LL (cm)	5.88	13.00	7.12	11.37	1.14	9.99
	LW (cm)	2.85	6.92	4.07	6.40	0.35	5.49
	L/W	0.95	2.06	1.11	1.78	0.16	8.89
	TL (cm)	7.25	97.17	89.92	36.40	21.55	59.20
	TD (mm)	1.75	9.25	7.50	6.55	2.09	31.91
	TFW (g)	5.60	707.00	701.40	341.43	183.91	53.87
Total	LL (cm)	11.30	19.27	5.88	13.39	3.46	30.60
	LW (cm)	6.91	11.73	2.85	8.88	1.73	25.03
	L/W	1.65	3.25	0.95	2.30	0.34	20.81
	TL (cm)	33.62	97.17	7.25	89.92	16.26	48.36
	TD (mm)	8.19	17.50	1.75	15.75	3.59	43.79
	TFW (g)	500.34	1,649.33	5.60	1,643.73	380.79	76.11

#### 3.1.2 Cluster analysis based on phenotypic traits

**FIGURE 3:**
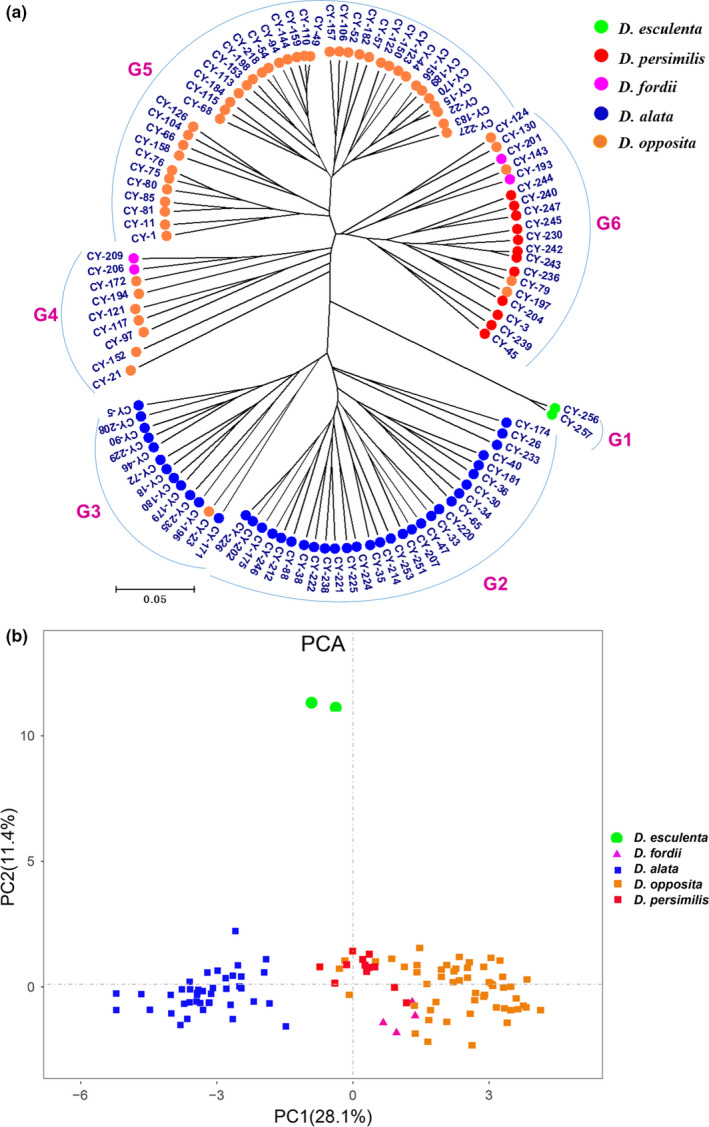
Cluster dendrogram (a) and PCA (b) of 112 yam accessions based on phenotypic traits. “G” stands for group

A cluster dendrogram of 112 yam accessions was created from 26 phenotypic traits (Figure 3a). The 112 accessions were divided into six major groups. The G1 group contained only two accessions of *D. esculenta*, CY‐256 and CY‐257, from Hainan Province, which were characterized by having no flowers, no aerial tubers, and no stem wings but having spines and twining anticlockwise. The G2 group consisted of 29 accessions belonging to *D. alata*, which were mainly characterized as being flowerless and having stem wings, a purple‐green stem, purple tuber flesh color or tuber skin color under bark, an nonaerial tubers, and a purple‐green petiole. The G3 group included 13 accessions and was very similar to the G2 group. The largest difference was that the tuber skin color under bark was mostly beige, while the tuber flesh color was mostly white. The accessions of the G2 and G3 groups belonged to *D. alata* (Figure 3a), and their common feature was the existence of stem wings. It is worth noting that CY‐23 of *D. opposita* was assigned to G3 because its phenotypic traits were similar to *D. alata* except for stem wings. In addition, the G2 group could be subdivided into three subgroups (Figure 3a). This further indicates that there is high diversity within *D. alata*. The G4 group contained nine accessions. CY‐206 and CY‐209 belonged to *D. fordii*, while the rest of the accessions belonged to *D. opposita*. The G5 group was composed of 40 accessions. The typical characteristics were flowers, aerial tubers, dark‐green leaf color, yellow‐green vein, cylindrical‐white tuber flesh color, and a length‐to‐width leaf ratio ranging from 1.2 to 1.5. The G6 group contained 19 accessions characterized by no flowers, no aerial tubers, oval leaves, purple petioles, yellow‐green veins, and brown tuber skin. *D. persimiis* was found only on G6, but *D. fordii* was found in the other groups (Figure 3a). *D. persimiis* was found only on G6, but *D. fordii* was found in the other groups. G4 and G5 were a group of *D. opposita*. The exception is the presence of one accession in G3 and a few in G6. This indicated that *D. fordii*, *D. persimilis,* and *D. opposita* had a close genetic relationship, and *D. opposita*, the most widely cultivated species in China, had high diversity.

Principal component analysis (PCA) was employed to analyze the phenotypic traits (Figure 3b). As shown, the two principal components (PCs) accounted for 28.1% (PC1) and 11.4% (PC2) of the total variance, respectively. The PC1 was dominated by flowering, aerial tubers, stem wing, tuber skin color, tuber skin color under bark, leaf length, leaf width, tuber length, and tuber diameter. The PC2 combined leaf shape, distance between lobes, leaf margin color, stem spine, twining direction, length‐to‐width ratio, and tuber flesh weight (Table S4). Similar to the cluster dendrogram, the 112 accessions were obviously divided into three groups. The accessions of *D. esculenta* and *D. alata* were separately clustered into one group, while a few accessions of *D. opposita*, the accessions of *D. fordii,* and the accessions of *D. persimilis* were very close together. This indicated that *D. esculenta* had a distant genetic relationship with other species, while *D. opposita*, *D. fordii,* and *D. persimilis* had a close genetic relationship.

### 3.2 Polymorphism analysis of molecular markers

#### 3.2.1 Marker efficiency analysis

A total of 104 bands and 99 polymorphic bands were amplified from 24 SSR primers, and the percentage of polymorphic loci was 95.19%. Ninety‐six pairs of alleles were amplified, and the largest *N*_a_ values (eight) were found using primers YM06 and YM08, with an average of four alleles per primer. The *N*_e_ values ranged from 1 to 6.429, with an average of 2.488. The mean *I*, observed heterozygosity (*H*_o_), and expected heterozygosity (*H*_e_) were 0.864, 0.422, and 0.441, respectively. The *H* values ranged from 0 to 0.845, with an average of 0.439 (Table 4). For species, the average *Na* of *D. opposita*, *D. alata*, *D. persimilis*, *D. fordii*, and *D. esculenta* were 3.583, 3.542, 2.25, 1.696, and 1.546, respectively. The *N*_r_ of *D. opposita* and *D. alata* was 1–8, *D. esculenta* was 1–3. Similar to *Na*, the highest for *I*, *H*_e_, and *H* was *D. opposita* and the lowest was *D. esculenta*, while the highest for *H_0_* was *D. persimilis* (Table 5).

**TABLE 4:** Genetic differentiation parameters of 112 yam accessions revealed by 24 polymorphic SSR markers

Marker	*N*_a_	*N*_e_	*I*	*H*_o_	*H*_e_	*H*
YM02	7	3.619	1.447	0.356	0.728	0.724
YM03	2	1.894	0.665	0.619	0.474	0.472
YM06	8	6.429	1.954	0.667	0.850	0.845
YM07	7	5.069	1.745	0.578	0.807	0.803
YM09	7	4.115	1.625	0.411	0.761	0.757
YM12	2	1.574	0.551	0.360	0.367	0.365
YM13	5	1.743	0.880	0.474	0.429	0.426
YM15	1	1	0	0	0	0
YM17	2	1.625	0.573	0.520	0.387	0.385
YM19	4	2.385	1.094	0.765	0.584	0.581
YM21	2	1.280	0.377	0.250	0.220	0.219
YM24	2	1.932	0.675	0.574	0.485	0.482
YM27	1	1	0	0	0	0
YM30	2	1.271	0.369	0.202	0.214	0.213
YM32	8	4.726	1.797	0.722	0.793	0.788
YM33	4	1.957	0.928	0.437	0.491	0.489
YM35	5	3.036	1.246	0.768	0.674	0.671
YM37	7	4.675	1.695	0.891	0.790	0.786
YM41	2	1.127	0.227	0.100	0.113	0.113
Dab2D08	2	1.032	0.081	0.0312	0.031	0.031
Da1A01	1	1	0	0	0	0
Da1D08	7	2.747	1.332	0.747	0.640	0.636
Da1F08	2	1.044	0.105	0	0.043	0.043
SSR−17	6	3.439	1.358	0.662	0.714	0.709
Mean	4	2.488	0.864	0.422	0.441	0.439
Total	96	59.717	20.724	10.134	10.593	10.535

Note

*N*_a_, observed number of alleles; *N*_e_, effective number of alleles; *I*, Shannon's diversity index; *H*_o_, observed heterozygosity; *H*_e_, expected heterozygosity; *H*, Nei's gene diversity.

**TABLE 5:** Genetic differentiation parameters of each species in yam revealed by 24 polymorphic SSR markers

Species	*N*_a_	*N*_r_	*N*_e_	*I*	*H*_o_	*H*_e_	*H*
*D. esculenta*	1.546	1–3	1.494	0.350	0.341	0.341	0.244
*D. fordii*	1.696	1–4	1.563	0.373	0.370	0.295	0.237
*D. alata*	3.542	1–8	2.281	0.789	0.398	0.418	0.412
*D. opposita*	3.583	1–8	2.462	0.795	0.440	0.419	0.414
*D. persimilis*	2.25	1–6	1.819	0.533	0.551	0.334	0.319

Note

*N*_a_, observed number of alleles; *N*_r_, range of observed number of alleles; *N*_e_, effective number of alleles; *I*, Shannon's diversity index; *H*_o_, observed heterozygosity; *H*_e_, expected heterozygosity; *H*, Nei's gene diversity.

From the 49 SRAP primers, 29 were selected because they produced distinct and stable bands, and 215 bands were amplified, of which 212 were polymorphic, accounting for 98.60% of the total bands. The number of bands amplified by each primer ranged from two to 11, with an average of 7.4. The polymorphic bands amplified by primers F1R2, F3R7, and F7R4 reached 11. The number of available alleles ranged from 1.072 to 1.519, with an average of 1.342, and the mean *H* value was 0.216. *I* varied from 0.145 to 0.472, with an average of 0.344 (Table 6). In terms of species, the number of polymorphic loci, *N*_a_, *N*_e_, *H*, and *I* of *D. alata* were the highest, while *D. fordii* were the lowest (Table 7).

**TABLE 6:** Genetic differentiation parameters of 112 yam accessions revealed by 29 polymorphic SRAP markers

Primer	Polymorphic loci	*N*_a_	*N*_e_	*H*	*I*
F1R1	8.00	2.00	1.332	0.205	0.322
F1R2	11.00	2.00	1.335	0.215	0.346
F1R3	4.00	2.00	1.495	0.291	0.435
F1R6	5.00	2.00	1.784	0.430	0.619
F2R1	5.00	2.00	1.335	0.241	0.402
F2R2	2.00	2.00	1.178	0.151	0.284
F2R6	8.00	2.00	1.197	0.138	0.237
F3R1	2.00	2.00	1.072	0.067	0.149
F3R2	9.00	2.00	1.332	0.200	0.319
F3R3	8.00	2.00	1.327	0.207	0.333
F3R6	7.00	2.00	1.346	0.231	0.378
F3R7	11.00	2.00	1.263	0.169	0.279
F4R2	7.00	2.00	1.395	0.252	0.400
F4R4	8.00	2.00	1.403	0.251	0.400
F4R5	8.00	2.00	1.519	0.312	0.472
F4R7	9.00	2.00	1.379	0.249	0.391
F5R1	8.00	2.00	1.233	0.137	0.220
F5R2	6.00	1.83	1.500	0.283	0.418
F5R3	6.00	1.83	1.497	0.285	0.423
F5R4	8.00	2.00	1.137	0.083	0.145
F5R6	10.00	2.00	1.404	0.267	0.426
F6R2	7.00	2.00	1.414	0.279	0.443
F6R3	5.00	2.00	1.250	0.156	0.256
F6R4	9.00	2.00	1.233	0.153	0.255
F6R5	8.00	2.00	1.198	0.134	0.226
F6R6	7.00	2.00	1.253	0.165	0.275
F7R1	9.00	2.00	1.440	0.272	0.424
F7R3	9.00	2.00	1.338	0.207	0.335
F7R4	11.00	1.91	1.344	0.219	0.353
Mean	7.40	1.99	1.342	0.216	0.344
Total	215.00	57.58	38.929	6.251	9.961

Note

*N*_a_, observed number of alleles; *N*_e_, effective number of alleles; *H*, Nei's gene diversity; *I*, Shannon's diversity index.

**TABLE 7:** Genetic differentiation parameters of each species in yam revealed by 29 polymorphic SRAP markers

Species	Polymorphic loci	Percentage of polymorphism	*N*_a_	*N*_e_	*H*	*I*
*D. esculenta*	63	29.30	1.293	1.207	0.121	0.177
*D. fordii*	47	21.86	1.219	1.138	0.080	0.119
*D. alata*	187	86.98	1.870	1.296	0.184	0.294
*D. opposita*	156	72.56	1.726	1.279	0.169	0.265
*D. persimilis*	148	68.84	1.688	1.250	0.158	0.255

Note

*N*_a_, observed number of alleles; *N*_e_, effective number of alleles; *H*, Nei's gene diversity; *I*, Shannon's diversity index.

#### 3.2.2 Cluster analysis based on SSR and SRAP markers

**FIGURE 4:**
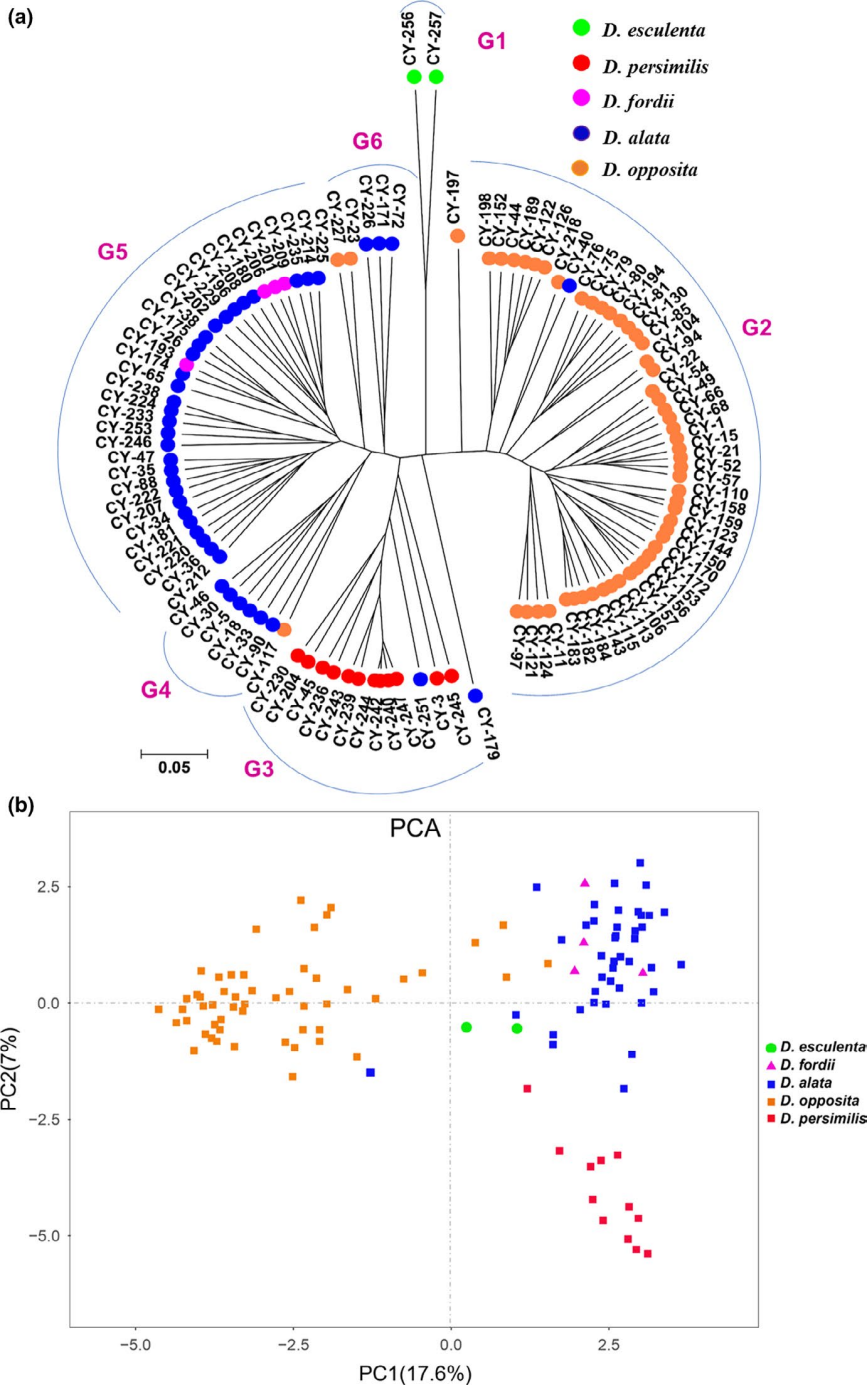
UPGMA cluster dendrogram (a) and PCA (b) of 112 yam accessions based on the combined SSR‐SRAP data. “G” is an abbreviation for group, and G1–G6 represents the different groups

Based on the polymorphic band data of SSR, SRAP, and SSR‐SRAP, cluster dendrogram analysis was performed, and the clustering results of SSR and SRAP markers were relatively consistent (Figure S1 and Figure 4a). Based on the cluster analysis of SSR data, the 112 accessions could be divided into four groups (Figure S1A). In general, the accessions of each species could be distinguished, but there were a few accessions of the same species that were not clustered together. Similar to the SSR analysis results, based on the cluster analysis of SRAP data, the 112 accessions could be divided into five groups, and there were also some accessions of species that were not distinguished from other accessions (e.g., CY‐256 and CY‐257 belonged to *D. esculenta*, Figure S1B). To this end, this study used SSR and SRAP polymorphic band data for joint cluster dendrogram analysis. As shown in Figure 4a, the 112 yam accessions were divided into six groups. The G1 group included CY‐256 and CY‐257 from Hainan Province, and both belonged to *D. esculenta*. A total of 51 accessions were assigned to the G2 group, with all except CY‐40 belonging to *D. opposita*. In addition, CY‐117 of the G4 group and CY‐227 and CY‐23 of the G6 group were also *D. opposita*. The accessions of *D. opposita* could be further divided into six subgroups. The G3 group contained 14 accessions, with CY‐179 and CY‐251 belonging to *D. alata*, and the remaining accessions belonging to *D. persimili*s. It is worth noting that CY‐240, CY‐242, CY‐244, and CY‐247 were closely related, indicating that they may belong to the same variety. Seven accessions were categorized into the G4 group, and the accession CY‐117 belonged to *D. opposita*, while the remaining belonged to *D. alata*. The G5 group was a collection of *D. fordii* and *D. alata*, in which 29 accessions were *D. alata*, and the remaining accessions (CY‐193, CY‐201, CY‐206, and CY‐209) were *D. fordii*. In the G6 group, CY‐227 and CY‐23 were *D. opposita*, and the remaining accessions were *D. alata*. From the clustering results, *D. alata* also had high diversity and could be further divided into four subgroups.

The results of PCA were basically consistent with those of molecular marker cluster analysis (Figure 4b). The 112 accessions could be divided into three groups. The accessions of *D. persimilis* and *D. opposita* could be separated separately, while the accessions of *D. alata* and *D. fordii* are clustered together.

#### 3.2.3 Population structure analysis

**FIGURE 5:**
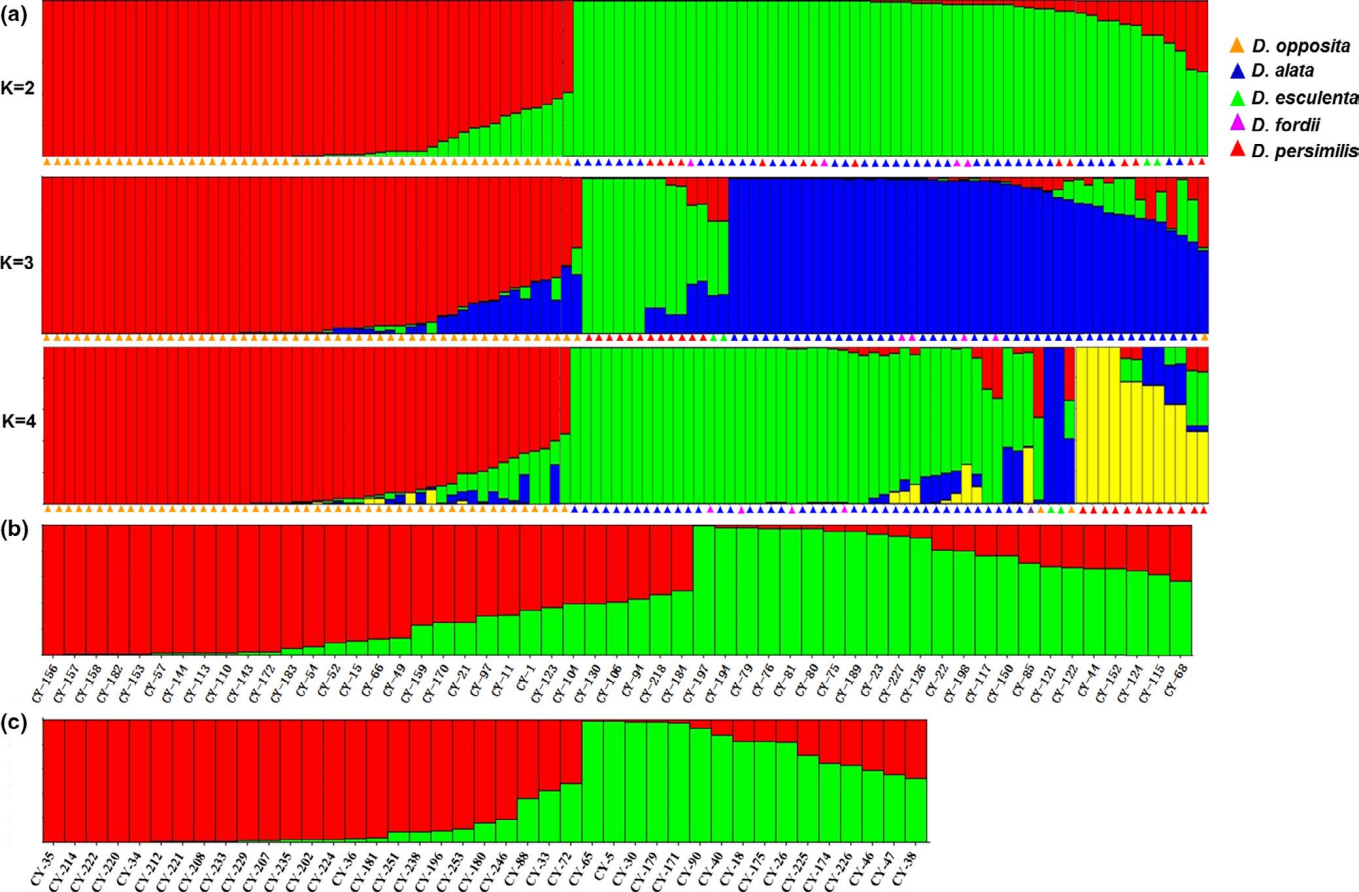
Population structure analysis of 112 yam accessions: (a) the population genetic structure of all accessions at *K* = 2, 3, 4; (b) *D. opposita* at *K* = 2 and (c) *D. alata* at *K* = 2

According to the output from STRUCTURE HARVESTER, when Δ*K* was at a maximum, the optimal *K* value was 2 (Figure S2A). At *K* = 2, the 112 accessions were divided into two subgroups, with red representing the first subgroup (51 accessions) and green representing the second subgroup (61 accessions). The first subgroup was a collection of all accessions of *D. opposita*, and the second subgroup was a collection of accessions of *D. alata*, *D. persimilis*, *D. fordii*, and *D. esculenta*. When *K* = 3, the accessions of *D. opposita* and *D. persimilis* were clustered into one group separately, whereas the other accessions were clustered into another group. At *K* = 4, the accessions of *D. alata* and *D. fordii* were mixed together to form a group, while the accessions of the other three species were grouped separately (Figure 5a). The genetic structure of *D. opposita* and *D. alata* was analyzed, respectively, and the *K* values of both Δ were the highest at *K* = 2 (Figure S2B,C), indicating that they could be divided into two subgroups. The two subgroups of *D. opposita* consist of 30 and 23 accessions, respectively. The first subgroup of *D. opposita* mainly contained accessions from three provinces of Shandong, Hebei, and Jiangsu, while second subgroup were from three provinces of Jiangxi, Sichuan, Henan (Figure 5b and Table S5). For *D. alata*, the first subgroup was composed of all accessions from Sichuan, most of Fujian and Jiangxi provinces, and the second subgroup was composed of all accessions from Yunnan, part of Jiangxi, and Jiangsu provinces (Figure 5c and Table S6).

## 4 DISCUSSION

### 4.1 Interspecific and intraspecific genetic differences in Yam landraces in China were discovered by combining phenotypic trait and molecular marker identification analyses

Phenotypic diversity is the external manifestation of genetic diversity, and it is the most basic method for germplasm selection and genetic background research (Mignouna, Dansi, et al., 2002; Mignouna, Mank, et al., 2002; Sartie et al., 2012; Zhang et al., 2019). In this study, 26 phenotypic traits of 112 yam accessions from five species were analyzed, and the results showed that the five species showed high diversity. Among the five species, the highest genetic diversity was found for *D. opposita*, followed by *D. alata*, *D. persimilis*, *D. fordii,* and *D. esculenta*.

The five species of yam showed high differentiation in different organs (leaf, stem, flower, aerial tubers, tuber, and root), and some phenotypic traits could be used for species identification. For instance, stem wings could be used effectively to identify *D. alata*, and stem spines and stem counterclockwise rotation could be used effectively to identify *D. esculenta*, which is consistent with previous reports (Bressan et al., 2011). Based on phenotypic variation, the 112 accessions were clustered into six groups, which was basically consistent with classical biological classification (Pei & Ding, 1985).

Additionally, flowering is a very important breeding requirement in any crop, but the entire genus *Dioscorea* is characterized by dioecy, and most important yam varieties are cultivated for their edible tubers and do not flower (Girma Tessema et al., 2017; Renner, 2014). In the present study, only 43 accessions (38.39%) flowered; two *D. fordii* accessions produced male flowers, while the remaining flowering accessions were *D. opposita*, 26 of which produced male flowers. The accessions of *D. esculenta*, *D. alata,* and *D. persimilis* did not flower. Previous research has shown that the flowering sex of yams is related to their yield. Tamiru et al. (2011) supposed that female yams mature early and produce tubers of excellent quality, but are less vigorous in growth compared to male yams and yield poorly under sub‐optimal conditions. It may be that the male flowers withered easily and had little influence on underground tubers, so their yield and quality were higher than those of female plants. This may explain why the majority of male flowers were observed in this study. Aerial tubers are an important organ of yam; they are also an effective means of nutritional reproduction and have been widely used in food or pharmaceutical applications (Asiedu & Sartie, 2010; Main et al., 2006). In this study, aerial tubers were found in two accessions of *D. fordii* and 39 accessions of *D. opposita*, but none were found in *D. alata*, *D. persimilis,* or *D. esculenta*. Thus, *D. opposita* and *D. fordii* might easily produce aerial tubers compared to other species.

Molecular markers have been widely used in the genetic diversity analysis of different species (Denwar et al., 2019; Saini et al., 2019; Yan et al., 2019). In this paper, 23 SSR and 29 SRAP primers were selected to analyze the genetic diversity of 112 accessions of 5 species of *Dioscorea*. The results showed that both markers had good amplification effects and could effectively and accurately capture the genetic variation of *Dioscorea*, and the polymorphism of SRAP markers was higher than that of SSR markers. SRAP markers target ORF regions (gene‐rich regions) and are dominant in nature (Wu et al., 2011), and SSR markers appear in both coding and noncoding regions and are codominant in nature (Tóth et al., 2000). The SSR markers amplified 96 alleles, with a mean observed number of alleles (*N*_a_) of 4 per marker locus, with a range of 1 to 7 alleles per marker locus, indicating the presence of haploids, diploids, triploids, tetraploids, pentaploids, and septaploids in the population. Sartie et al. (2012) also obtained similar results regarding the genetic and phenotypic diversity of a tropical yam germplasm collection. Moreover, Wu et al. (2019) reported a lower number of alleles in *D. alata* (*N*_a_ = 1.810, *N*_e_ = 1.470), while Loko et al. (2016) reported a higher number of alleles in Guinea yam (*N*_a_ = 8.69). Nei's (1973) gene diversity (*H* = 0.439) in this study was higher than that in Siqueira et al. (2014) reported for Brazilian *D. alata* (*H* = 0.410) but lower than that in Tostain et al. (2007) reported for *D. rotundata* (*H* = 0.500). In addition, the observed heterozygosity (0.422) was close to the expected heterozygosity (0.441), indicating that *Dioscorea* crops had a high level of genetic diversity (Sewall, 1978). For SRAP marker analysis, the detected Shannon's diversity index (*I* = 0.344), Nei's (1973) gene diversity (*H* = 0.216), the observed number of alleles (*N*_a_=1.990), and the effective number of alleles (*N*_e_=1.342) were also similar to the results of Li et al. (21, *I* = 0.368, *H* = 0.246, *N*_a _= 1.742, *N*_e_ = 1.322) and Wu (2013, *I* = 0.442, *H* = 0.288).

Furthermore, the cluster analysis of 112 Chinese yam landraces based on the polymorphic band data of molecular markers was in good agreement with the cluster analysis of phenotypic traits. The difference between the two analyses was that the accessions of *D. opposita* and *D. persimilis* could be successfully identified by molecular markers, while the accessions of *D. fordii* and *D. alata* could be identified by phenotypic traits. This may explain why the number of phenotypic traits is limited, and some traits are ignored, leading to insufficient analysis. This may also be due to the incomplete genomic information of yam. As a result of the lack of molecular markers closely related to phenotypic traits and the application of molecular markers in different populations of yam, genetic maps cannot be integrated with each other, resulting in low density and poor universality. The above may be the reason for the difference between molecular markers and phenotypic traits. Thus, the combined analysis of the two methods can identify the landraces of yam well (Denwar et al., 2019; Siqueira et al., 2014). For instance, CY‐3 (ZhuGaoShu) is a native variety that has been cultivated for 500 years in Jiangxi Province; its leaves look similar to those of *D. persimilis*, but the tuber grows similar to that of *D. opposita*, the species designation is not clear. “ZhuGaoShu” yam is now identified as *D. persimilis* based on our results. Furthermore, we collected CY‐76, CY‐79, CY‐80, CY‐81, and CY‐194 in Ruicheng County, Jiangxi Province; these accessions were previously thought to be *D. opposita*, but their phenotypic characteristics were different from those of this species. By combining our results with chloroplast genome sequencing analysis (data not shown), we preliminarily speculated that this group may be a new species or a new variant of *D. opposita*.

### 4.2 The possible origin and domestication of *D. opposita* and *D. alata* were speculated by genetic differences and population structure analysis of Chinese yam resources

At present, the origin of yam can be traced back to the Late Cretaceous (Maurin et al., 2016). *Dioscorea* is considered to be a monophyletic group originating from a common ancestor (Wu et al., 2014), which represents an early‐diverging lineage of monocots just internal to *Acorus* (Hansen et al., 2007). However, there are still many different arguments about the origin, evolution process, and domestication process of *Dioscorea*.

In this study, a total of 112 cultivars, landraces, and wild varieties of yam were collected in 21 provinces (cities) in China, and their genetic diversity was comprehensively evaluated. *D. opposita* is a well‐known vegetable; it has the largest cultivated area and is the most widely distributed yam species in China. *D. opposita* has been used as food and traditional medicine in China for thousands of years (Amat et al., 2014), but it is almost unknown to the rest of the world. *D. opposita* was distributed in Heilongjiang Province in the north, Hainan Province in the south, Shanghai in the east, and Sichuan Province in the west. Its growth environment was also more complicated, including plains, mountainous regions, and coastal areas. Therefore, this may be the reason for the larger genetic diversity compared with other species, as these plants had to adapt to their respective growth environments, climate change, and climatic conditions. Compared with other species, *D. opposita* had the shortest growth period and the smallest leaves, indicating that its aboveground biomass was smaller. Based on these phenotypic characteristics, it is speculated that *D. opposita* may originate in the temperate zone, and it is also considered to be the only edible yam species that can be grown in the temperate zone (Epping & Laibach, 2020). In addition, a large number of wild resources of *D. opposita* were distributed in the northern and southern provinces of China. In the present study, five wild resources were collected in Henan (CY‐117, CY‐150), Hebei (CY‐94), Guizhou (CY‐104), Sichuan (CY‐124), and Jiangxi (CY‐153) provinces. This may be evidence that *D. opposita* may have originated in China and been domesticated from wild species.

Forty‐one accessions of *D. alata* were collected in eight provinces in Southern China, accounting for 36.61% of the total samples. The most typical features of this species were stem wings, strong growth potential, and a long growth period, and these characteristics were similar to previous results (Bressan et al., 2011). This may be evidence that *D. alata* may have originated in tropical or subtropical areas. With regard to a previous report, *D. alata* may have originated in the north and east of the Bay of Bengal and spread to Southeast Asia, Malaysia, Pacific tropical islands, Africa, and America (Nemorin et al., 2013). Some authors have proposed that *D. alata* was domesticated in India or Yunnan Province in China (Coursey, 1976; Chaïr et al., 2005; Wu et al., 2019). Sharif et al. (2020) explored the geographical diversification and dispersal of the polyploid and clonally propagated *D. alata* by sequencing 643 accessions, and their findings support the hypothesis of independent domestication origins in two major gene pools in Asia and the Pacific. Moreover, *D. alata* is considered a heterozygous species and may have resulted from a cross between the wild relatives *D. hamiltonii* and *D. persimilis* (Nemorin et al., 2013).

The wild resources of *D. persimilis* were previously reported to be distributed in Hunan, Guangdong, Guangxi, Guizhou, and Yunnan provinces (Pei & Ding, 1985). We also collected accessions of *D. persimilis* in Fujian and Jiangxi provinces, which have a long history of yam cultivation. In addition, *D. persimilis* and *D. opposita* were closely related, and some accessions were highly similar based on phenotypic traits. The rDNA internal transcribed spacer (ITS) sequences also showed that *D. persimilis* and *D. opposita* were closely related (Liu et al., 2001; Wu et al., 2013). It has also been speculated that *D. persimilis* is mutant form of *D. opposita* (Liu et al., 2001). The wild resources of *D. fordii* are distributed in Zhejiang, Guangdong, Guangxi, Fujian, and Hunan provinces. This species has been widely cultivated for more than 200 years for its high yield and good resistance to stress. *D. fordii* may have formed from long‐term domestication of wild species. In the current study, CY‐193, CY‐201, CY‐206, and CY‐209 from *D. fordii* were grouped with *D. alata* (Figure 3a,b), indicating that *D. alata* and *D. fordii* were the closest species genetically. Similar results were obtained by Lei et al. (2013) and Li et al. (2016) based on genetic diversity and Wu et al. (2014) based on ITS data. However, future research on the origin and evolution of *Dioscorea* requires additional genomic information and an increased number of species.

## 5 CONCLUSION

The germplasm of yam species widely used in cultivation shows high intraspecific and interspecific diversity in China. Phenotypic and molecular markers are very effective tools to detect the diversity of yam. The best method to identify genetic differences is combining molecular and phenotypic data to obtain more information for genetic relationship clarification. The results of cluster analysis showed that *D. esculenta* had the farthest genetic relationship with other species, while *D. alata, D. fordii, D. persimilis*, and *D. opposita* had the closest genetic relationship, and PCA and population structure analyses further confirmed this result. In addition, the analysis of the origin and genetic relationship of yam indicated that *D. opposita* may have originated in China. In summary, the results of this study provide a theoretical basis for identifying the genetic differences and resource types of yam landraces in China.

## CONFLICT OF INTEREST

The authors declare that they have no conflicts of interest.

## AUTHOR CONTRIBUTIONS

**Tianxu Cao:** Formal analysis (equal); Investigation (equal); Methodology (equal); Writing‐original draft (equal). **Jingyu Sun:** Methodology (equal). **Nan Shan:** Formal analysis (equal). **Xin Chen:** Formal analysis (equal). **Putao Wang:** Formal analysis (equal); Supervision (equal). **Qianglong Zhu:** Formal analysis (equal). **Yao Xiao:** Writing‐review & editing (supporting). **Hongyu Zhang:** Investigation (equal). **Qinghong Zhou:** Formal analysis (equal); Project administration (equal); Writing‐review & editing (equal). **Yingjin Huang:** Project administration (equal); Supervision (equal); Writing‐review & editing (equal).

## Supporting information

Supplementary Material

## Data Availability

Supplementary data sets are available at the associated Dryad repository: https://doi.org/10.5061/dryad.gmsbcc2kw.
