# Supplementary material for: Uncovering the genetic diversity of yams (Dioscorea spp.) in China by combining phenotypic trait and molecular marker analyses
Source: Ecol Evol. 2021 Jul 16;11(15):9970–86. doi: 10.1002/ece3.7727 (PMC8328405; doi:10.1002/ece3.7727)
Supplement: Supplementary file 1 — Supplementary Material [file ECE3-11-9970-s001.docx]

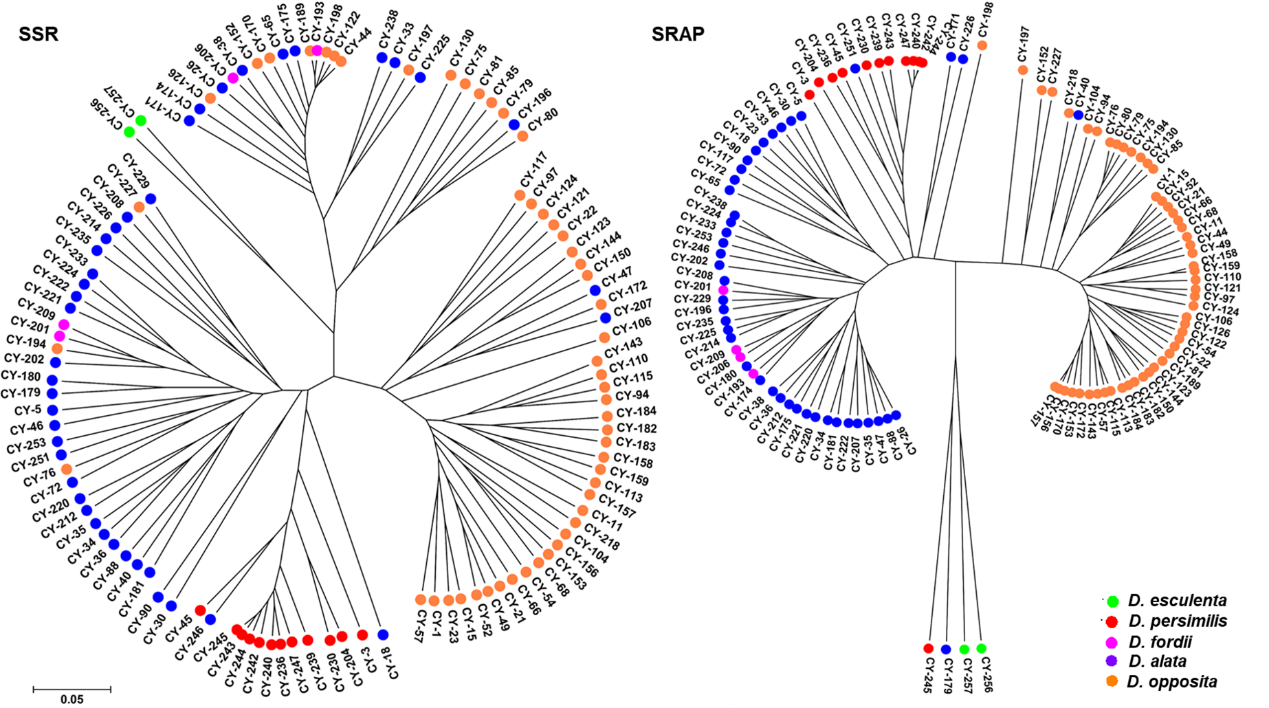


Supplementary Fig. 1: UPGMA cluster dendrogram of 112 yam accessions based on SSR and SRAP data, respectively.


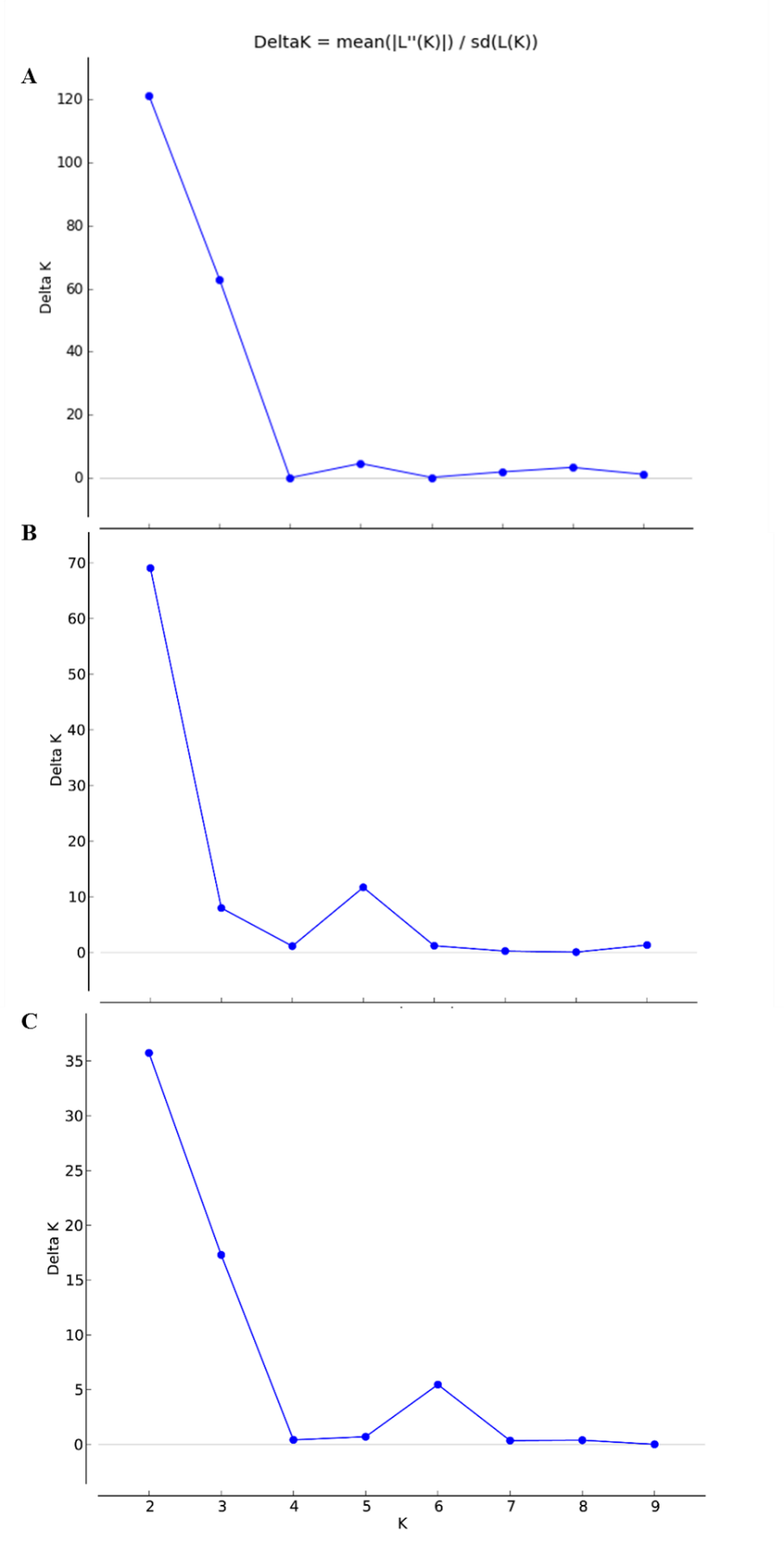


Supplementary Fig. 2 Population structure analysis ΔK values for different numbers of populations assumed (K) of all accessions (A), *D. opposita* (B) and *D. alata* (C).

Supplementary Table 1 List of yam accessions and their origin.

| Code | Sequencing NO. | Accession name | Type | Origin | species |
| --- | --- | --- | --- | --- | --- |
| 1 | CY-88 | Jinzhai L1 | Landrace | Anhui | *D. alata* |
| 2 | CY-68 | Fulin L1 | Landrace | Chongqing | *D.opposita* |
| 3 | CY-175 | Fujian L8 | Landrace | Fujian | *D. alata* |
| 4 | CY-182 | June Yam | Landrace | Fujian | *D.opposita* |
| 5 | CY-202 | Minghuai 1 | Landrace | Fujian | *D. alata* |
| 6 | CY-204 | Yangmingshan Yam | Landrace | Fujian | *D.persimilis* |
| 7 | CY-220 | Changting L2 | Landrace | Fujian | *D. alata* |
| 8 | CY-221 | Sanming L7 | Landrace | Fujian | *D. alata* |
| 9 | CY-222 | Jianning L1 | Landrace | Fujian | *D. alata* |
| 10 | CY-224 | Nanping L2 | Landrace | Fujian | *D. alata* |
| 11 | CY-225 | Nanping L1 | Landrace | Fujian | *D. alata* |
| 12 | CY-229 | Fuzhou L1 | Landrace | Fujian | *D. alata* |
| 13 | CY-230 | Sanming L1 | Landrace | Fujian | *D.persimilis* |
| 14 | CY-236 | Mingxi Yam | Landrace | Fujian | *D.persimilis* |
| 15 | CY-238 | Minghuai 3 | Landrace | Fujian | *D. alata* |
| 16 | CY-239 | Qingliu Yam | Landrace | Fujian | *D.persimilis* |
| 17 | CY-240 | Sanming L2 | Landrace | Fujian | *D.persimilis* |
| 18 | CY-242 | Sanming L3 | Landrace | Fujian | *D.persimilis* |
| 19 | CY-243 | Sanming L4 | Landrace | Fujian | *D.persimilis* |
| 20 | CY-244 | Sanming L5 | Landrace | Fujian | *D.persimilis* |
| 21 | CY-245 | Sanming L6 | Landrace | Fujian | *D.persimilis* |
| 22 | CY-247 | Shangge Yam | Landrace | Fujian | *D.persimilis* |
| 23 | CY-251 | Zhouning purple Yam | Landrace | Fujian | *D. alata* |
| 24 | CY-201 | Guangxi L1 | Landrace | Guangxi | *D. fordii* |
| 25 | CY-206 | Guihuai 2 | Landrace | Guangxi | *D. fordii* |
| 26 | CY-209 | Guihuai 5 | Landrace | Guangxi | *D. fordii* |
| 27 | CY-193 | Nancheng L2 | Landrace | Jiangxi | *D. fordii* |
| 28 | CY-226 | Zengcheng L1 | Landrace | Guangdong | *D. alata* |
| 29 | CY-104 | Zunyi W1 | Wild | Guizhou | *D.opposita* |
| 30 | CY-11 | Hainan L1 | Landrace | Hainan | *D.opposita* |
| 31 | CY-256 | Maoshu 61 | Landrace | Hainan | *D. esculenta* |
| 32 | CY-257 | Maoshu 70 | Landrace | Hainan | *D. esculenta* |
| 33 | CY-156 | Stick Yam | Landrace | Hebei | *D.opposita* |
| 34 | CY-158 | Xiaobaizui Yam | Landrace | Hebei | *D.opposita* |
| 35 | CY-49 | Anping white yam | Landrace | Hebei | *D.opposita* |
| 36 | CY-52 | Hebei Ma Yam | Landrace | Hebei | *D.opposita* |
| 37 | CY-94 | Jinzhou W1 | Wild | Hebei | *D.opposita* |
| 38 | CY-117 | Xiuwu W1 | Wild | Henan | *D.opposita* |
| 39 | CY-121 | Dancheng L1 | Landrace | Henan | *D.opposita* |
| 40 | CY-123 | Boai L1 | Landrace | Henan | *D.opposita* |
| 41 | CY-150 | Boai W1 | Wild | Henan | *D.opposita* |
| 42 | CY-44 | Iron stick Yam L1 | Landrace | Henan | *D.opposita* |
| 43 | CY-54 | Iron stick Yam L2 | Landrace | Henan | *D.opposita* |
| 44 | CY-97 | Lushan L1 | Landrace | Henan | *D.opposita* |
| 45 | CY-115 | Alpine red yam | Landrace | Hubei | *D.opposita* |
| 46 | CY-130 | Wuxue L1 | Landrace | Hubei | *D.opposita* |
| 47 | CY-15 | Yongchuan L1 | Landrace | Hubei | *D.opposita* |
| 48 | CY-157 | Shuangbao Yam | Landrace | Jiangsu | *D.opposita* |
| 49 | CY-171 | Chenggan Yam | Landrace | Jiangsu | *D. alata* |
| 50 | CY-172 | Meidai Yam | Landrace | Jiangsu | *D.opposita* |
| 51 | CY-174 | Xuzhou Pink Yam | Landrace | Jiangsu | *D. alata* |
| 52 | CY-179 | Suyu 6 | Landrace | Jiangsu | *D. alata* |
| 53 | CY-180 | Suyu 7 | Landrace | Jiangsu | *D. alata* |
| 54 | CY-181 | Suyu 1 | Landrace | Jiangsu | *D. alata* |
| 55 | CY-183 | Suyu 7 | Landrace | Jiangsu | *D.opposita* |
| 56 | CY-184 | Fruit Yam | Landrace | Jiangsu | *D.opposita* |
| 57 | CY-207 | Suzi 1 | Landrace | Jiangsu | *D. alata* |
| 58 | CY-218 | Mudanjiang L1 | Landrace | Heilongjiang | *D.opposita* |
| 59 | CY-1 | Yongfeng Yam | Landrace | Jiangxi | *D.opposita* |
| 60 | CY-153 | Pingxiang W1 | Wild | Jiangxi | *D.opposita* |
| 61 | CY-194 | Ruichang L7 | Landrace | Jiangxi | *D.opposita* |
| 62 | CY-214 | Quannan L2 | Landrace | Jiangxi | *D. alata* |
| 63 | CY-227 | Nancheng L1 | Landrace | Jiangxi | *D.opposita* |
| 64 | CY-233 | Ganzi 1 | Landrace | Jiangxi | *D. alata* |
| 65 | CY-26 | Anyuan L1 | Landrace | Jiangxi | *D. alata* |
| 66 | CY-3 | Taihe Penny's Yam | Landrace | Jiangxi | *D.persimilis* |
| 67 | CY-30 | Anyuan L5 | Landrace | Jiangxi | *D. alata* |
| 68 | CY-33 | Yudu L3 | Landrace | Jiangxi | *D. alata* |
| 69 | CY-34 | Yudu L4 | Landrace | Jiangxi | *D. alata* |
| 70 | CY-35 | Yudu L5 | Landrace | Jiangxi | *D. alata* |
| 71 | CY-36 | Yudu L6 | Landrace | Jiangxi | *D. alata* |
| 72 | CY-38 | Yudu L8 | Landrace | Jiangxi | *D. alata* |
| 73 | CY-40 | Yudu L10 | Landrace | Jiangxi | *D. alata* |
| 74 | CY-45 | Ruijin L1 | Landrace | Jiangxi | *D.persimilis* |
| 75 | CY-46 | Ruijin L2 | Landrace | Jiangxi | *D. alata* |
| 76 | CY-47 | Ruijin L3 | Landrace | Jiangxi | *D. alata* |
| 77 | CY-5 | Xingguo L1 | Landrace | Jiangxi | *D. alata* |
| 78 | CY-65 | Quannan L1 | Landrace | Jiangxi | *D. alata* |
| 79 | CY-72 | Fulin L2 | Landrace | Jiangxi | *D. alata* |
| 80 | CY-75 | Ruichang L1 | Landrace | Jiangxi | *D.opposita* |
| 81 | CY-76 | Ruichang L2 | Landrace | Jiangxi | *D.opposita* |
| 82 | CY-79 | Ruichang L3 | Landrace | Jiangxi | *D.opposita* |
| 83 | CY-80 | Ruichang L4 | Landrace | Jiangxi | *D.opposita* |
| 84 | CY-81 | Ruichang L5 | Landrace | Jiangxi | *D.opposita* |
| 85 | CY-85 | Ruichang L6 | Landrace | Jiangxi | *D.opposita* |
| 86 | CY-21 | Fusong 1 | Landrace | Jilin | *D.opposita* |
| 87 | CY-22 | Fusong 2 | Landrace | Jilin | *D.opposita* |
| 88 | CY-23 | Fusong 3 | Landrace | Jilin | *D.opposita* |
| 89 | CY-126 | Bayan L1 | Landrace | Neimenggu | *D.opposita* |
| 90 | CY-106 | Baiyu Yam | Landrace | Shandong | *D.opposita* |
| 91 | CY-110 | Jinxiang L1 | Landrace | Shandong | *D.opposita* |
| 92 | CY-122 | Chenji Tiegun Yam | Landrace | Shandong | *D.opposita* |
| 93 | CY-143 | Niutui Yam | Landrace | Shandong | *D.opposita* |
| 94 | CY-144 | Jipicao Yam | Landrace | Shandong | *D.opposita* |
| 95 | CY-159 | Ximaochang Yam | Landrace | Shandong | *D.opposita* |
| 96 | CY-57 | Huai Yam | Landrace | Shandong | *D.opposita* |
| 97 | CY-66 | Ximaochang Yam | Landrace | Shandong | *D.opposita* |
| 98 | CY-189 | Pingyao Yam | Landrace | Shanxi | *D.opposita* |
| 99 | CY-113 | Suining L1 | Landrace | Sichuan | *D.opposita* |
| 100 | CY-124 | Jinniu W1 | Wild | Sichuan | *D.opposita* |
| 101 | CY-196 | Tianquan L1 | Landrace | Sichuan | *D. alata* |
| 102 | CY-197 | Baoxing L2 | Landrace | Sichuan | *D.opposita* |
| 103 | CY-198 | Baoxing L1 | Landrace | Sichuan | *D.opposita* |
| 104 | CY-208 | Yashanyao L1 | Landrace | Sichuan | *D. alata* |
| 105 | CY-212 | Miyi L2 | Landrace | Sichuan | *D. alata* |
| 106 | CY-235 | Miyi L4 | Landrace | Sichuan | *D. alata* |
| 107 | CY-253 | Miyizihua Yam | Landrace | Sichuan | *D. alata* |
| 108 | CY-246 | Miyi L1 | Landrace | Taiwan | *D. alata* |
| 109 | CY-152 | Honglong Yam | Landrace | Yunnan | *D.opposita* |
| 110 | CY-18 | Honghe L2 | Landrace | Yunnan | *D. alata* |
| 111 | CY-90 | Nuomi Yam | Landrace | Yunnan | *D. alata* |
| 112 | CY-170 | Wenke 4 | Landrace | Zhejiang | *D.opposita* |

Supplementary Table 2 List of the 24 SSR markers selected.

| Prime name | Forward sequence (5′-3′) | Reverse primer sequence (3′-5′) |
| --- | --- | --- |
| YM02 | TAGATTTCGCTTTTCCACTAGC | CCTAATCATCATCATCGTCATC |
| YM03 | TCACTCAAACAATGAGCGTAG | GATGGCTGCTGCATGACTG |
| YM06 | ACAGAGCTGTTGACACAAACA | CCTCAATGAACCTTTGGTCTA |
| YM07 | AGCATTGGGTCCTTTCATCC | ACAATTCACACAAAGCATGGC |
| YM09 | AGGAACATTCCCACTCAGTTATG | ATTGGGCAAGTGTGGTGTG |
| YM12 | TGAGCATTCTTGTTTTGCCG | CTTTCAGGGCGTGCATGG |
| YM13 | CCAATCACATCACGTCTAGTCT | GACAATAGAAACTTCGAGACCC |
| YM15 | CCATCTCCTCCCTTATCTACAC | GGGATTGAAGTTCCAGAGACTA |
| YM17 | TCCCTCAATTAAAGCATAGCCTC | AGCCACCAAACATCTTGCTC |
| YM19 | ACGGAAGCAGCAAGAGGAG | GTGTCATCAGCATCTGGGC |
| YM21 | AATGATGCATCTGAGGATAGTG | GATGCTATTACGACAACCTTGA |
| YM24 | GGTGTTGTTGGGTTTCATTGTC | TCCCTCTTCTCATTTCACTCCC |
| YM27 | TCCAGCTCTTTAGCACAGG | AGGAGCATAGGCAACAAGC |
| YM30 | CCACAACTAAAAACACATGGAC | GTGGTAGGGTGTGTAGCTTCTT |
| YM32 | GAGGTCTGCGACGGATTTG | TCGCATTCTTCATCCTCTTCAC |
| YM33 | ACCATGGGATGAAGGGAAGG | GCATATGGTGCATGGGAGC |
| YM35 | GCTCTAGCAAACAATCCAATC | CCCTATACGCATGAAAGTAACA |
| YM37 | GCCTTGTTTTGTTGATGCTTCG | CCAGCCCACTAATCCCTCC |
| YM41 | GCCTTGTTTTGTTGATGCTTCG | CCAGCCCACTAATCCCTCC |
| Da1A01 | TATAATCGGCCAGAGG | TGTTGGAAGCATAGAGAA |
| Da1D08 | GATGCTATGAACACAACTAA | TTTGACAGTGAGAATGGA |
| Da1F08 | AATGCTTCGTAATCCAAC | CTATAAGGAATTGGTGCC |
| Dab2D08 | GATGCTATGAACACAACTAA | TTTGACAGTGAGAATGGA |
| ssr-17 | GCAACTGTCCTACTCCGACC | TGCTGCCTCAAGTTCCACAA |

Supplementary Table 3 Sequences of SRAP primers.

| Prime name | Forward sequence (5′-3′) | Prime name | Reverse primer sequence (3′-5′) |
| --- | --- | --- | --- |
| Me6 | TGAGTCCAAACCGGACA | Em6 | GACTGCGTACGAATTGCA |
| Me8 | TGAGTCCAAACCGGACT | Em8 | GACTGCGTACGAATTCAC |
| Me9 | TGAGTCCAAACCGGAGG | Em9 | GACTGCGTACGAATTCAG |
| Me10 | TGAGTCCAAACCGGAAA | Em10 | GACTGCGTACGAATTCAT |
| Me11 | TGAGTCCAAACCGGAAC | Em11 | GACTGCGTACGAATTCTA |
| Me12 | TGAGTCCAAACCGGAGA | Em12 | GACTGCGTACGAATTCTC |
| Me13 | TGAGTCCAAACCGGAAG | Em13 | GACTGCGTACGAATTCTG |

Supplementary Table 4 The eigenvalues of PC1 and PC2 based on principal component analysis of phenotypic traits.

| Traits | PC1 | PC2 |
| --- | --- | --- |
| FL | 0.269 | -0.039 |
| AT | 0.277 | -0.021 |
| LS | 0.183 | -0.220 |
| LC | 0.178 | -0.098 |
| LAX | -0.058 | -0.139 |
| DBL | 0.086 | -0.335 |
| LMC | 0.071 | -0.246 |
| PC | -0.070 | -0.017 |
| LVC | -0.035 | -0.157 |
| LV | -0.049 | 0.110 |
| SW | -0.334 | -0.091 |
| SC | 0.191 | -0.030 |
| SSP | -0.012 | 0.500 |
| TD | -0.012 | 0.500 |
| TS | -0.309 | 0.068 |
| RHD | 0.053 | -0.030 |
| PRT | -0.076 | -0.072 |
| TSC | 0.210 | 0.108 |
| TSCUB | -0.283 | -0.078 |
| FC | -0.252 | -0.095 |
| LL | -0.319 | -0.075 |
| LW | -0.240 | 0.130 |
| L/W | -0.180 | -0.232 |
| TL | 0.230 | -0.191 |
| TD | -0.225 | -0.100 |
| TFW | -0.162 | -0.207 |

Supplementary Table 5 Population genetic structure analysis of *D. opposita.*

| First subgroup | | | | Second subgroup | | | |
| --- | --- | --- | --- | --- | --- | --- | --- |
| Seq. NO. | species | Accession name | Origin | Seq. NO. | species | Accession name | Origin |
| CY-182 | *D.opposita* | June Yam | Fujian | CY-68 | *D.opposita* | Iron stick Yam | Chongqing |
| CY-104 | *D.opposita* | Zunyi W1 | Guizhou | CY-117 | *D.opposita* | Xiuwu W1 | Henan |
| CY-11 | *D.opposita* | Hainan L1 | Hainan | CY-121 | *D.opposita* | Dancheng L1 | Henan |
| CY-156 | *D.opposita* | Stick Yam | Hebei | CY-150 | *D.opposita* | Boai W1 | Henan |
| CY-158 | *D.opposita* | Xiaobaizui Yam | Hebei | CY-44 | *D.opposita* | Iron stick Yam | Henan |
| CY-49 | *D.opposita* | Anping white yam | Hebei | CY-115 | *D.opposita* | Alpine red yam | Hubei |
| CY-52 | *D.opposita* | Hebei Ma Yam | Hebei | CY-194 | *D.opposita* | Ruichang L7 | Jiangxi |
| CY-94 | *D.opposita* | Jinzhou W1 | Hebei | CY-227 | *D.opposita* | Nancheng L1 | Jiangxi |
| CY-218 | *D.opposita* | Mudanjiang L1 | Heilongjiang | CY-75 | *D.opposita* | Ruichang L1 | Jiangxi |
| CY-123 | *D.opposita* | Boai L1 | Henan | CY-76 | *D.opposita* | Ruichang L2 | Jiangxi |
| CY-54 | *D.opposita* | Iron stick Yam | Henan | CY-79 | *D.opposita* | Ruichang L3 | Jiangxi |
| CY-97 | *D.opposita* | Lushan L1 | Henan | CY-80 | *D.opposita* | Ruichang L4 | Jiangxi |
| CY-130 | *D.opposita* | Wuxue L1 | Hubei | CY-81 | *D.opposita* | Ruichang L5 | Jiangxi |
| CY-15 | *D.opposita* | Yongchuan L1 | Hubei | CY-85 | *D.opposita* | Ruichang L6 | Jiangxi |
| CY-157 | *D.opposita* | Shuangbao Yam | Jiangsu | CY-22 | *D.opposita* | Fusong 2 | Jilin |
| CY-172 | *D.opposita* | Meidai Yam | Jiangsu | CY-23 | *D.opposita* | Fusong 3 | Jilin |
| CY-183 | *D.opposita* | Suyu 7 | Jiangsu | CY-126 | *D.opposita* | Bayan L1 | Neimenggu |
| CY-184 | *D.opposita* | Fruit Yam | Jiangsu | CY-122 | *D.opposita* | Chenji Tiegun Yam | Shandong |
| CY-1 | *D.opposita* | Yongfeng Yam | Jiangxi | CY-189 | *D.opposita* | Pingyao Yam | Shanxi |
| CY-153 | *D.opposita* | Pingxiang W1 | Jiangxi | CY-124 | *D.opposita* | Jinniu W1 | Sichuan |
| CY-21 | *D.opposita* | Fusong 1 | Jilin | CY-197 | *D.opposita* | Baoxing 2 | Sichuan |
| CY-106 | *D.opposita* | Baiyu Yam | Shandong | CY-198 | *D.opposita* | Baoxing 1 | Sichuan |
| CY-110 | *D.opposita* | Jinxiang L1 | Shandong | CY-152 | *D.opposita* | Honglong Yam | Yunnan |
| CY-143 | *D.opposita* | Niutui Yam | Shandong |  |  |  |  |
| CY-144 | *D.opposita* | Jipicao Yam | Shandong |  |  |  |  |
| CY-159 | *D.opposita* | Ximaochang Yam | Shandong |  |  |  |  |
| CY-57 | *D.opposita* | Huai Yam | Shandong |  |  |  |  |
| CY-66 | *D.opposita* | Ximaochang Yam | Shandong |  |  |  |  |
| CY-113 | *D.opposita* | Suining L1 | Sichuan |  |  |  |  |
| CY-170 | *D.opposita* | Wenke 4 | Zhejiang |  |  |  |  |

Supplementary Table 6 Population genetic structure analysis of *D. alata*.

| First subgroup | | | | Second subgroup | | | |
| --- | --- | --- | --- | --- | --- | --- | --- |
| Seq. NO. | species | Accession name | Origin | Seq. NO. | species | Accession name | Origin |
| CY-180 | *D. alata* | Suyu 7 | Jiangsu | CY-171 | *D. alata* | Chenggan Yam | Jiangsu |
| CY-181 | *D. alata* | Suyu 1 | Jiangsu | CY-175 | *D. alata* | Fujian W1 | Fujian |
| CY-88 | *D. alata* | Jinzhai L1 | Anhui | CY-225 | *D. alata* | Nanping L1 | Fujian |
| CY-202 | *D. alata* | Minghuai 1 | Fujian | CY-226 | *D. alata* | Zengcheng L1 | Guangzhou |
| CY-220 | *D. alata* | Changting L2 | Fujian | CY-174 | *D. alata* | Xuzhou Pink Yam | Jiangsu |
| CY-221 | *D. alata* | Sanming L7 | Fujian | CY-179 | *D. alata* | Suyu 6 | Jiangsu |
| CY-222 | *D. alata* | Jianning L1 | Fujian | CY-26 | *D. alata* | Anyuan L1 | Jiangxi |
| CY-224 | *D. alata* | Nanping L2 | Fujian | CY-30 | *D. alata* | Anyuan L5 | Jiangxi |
| CY-229 | *D. alata* | Fuzhou L1 | Fujian | CY-38 | *D. alata* | Yudu L8 | Jiangxi |
| CY-238 | *D. alata* | Minghuai 3 | Fujian | CY-40 | *D. alata* | Yudu L10 | Jiangxi |
| CY-251 | *D. alata* | Zhouning purple Yam | Fujian | CY-46 | *D. alata* | Ruijin 2 | Jiangxi |
| CY-207 | *D. alata* | Suzi 1 | Jiangsu | CY-47 | *D. alata* | Ruijin L3 | Jiangxi |
| CY-214 | *D. alata* | Quannan L2 | Jiangxi | CY-5 | *D. alata* | Xingguo L1 | Jiangxi |
| CY-233 | *D. alata* | Ganzi 1 | Jiangxi | CY-65 | *D. alata* | Quannan L1 | Jiangxi |
| CY-33 | *D. alata* | Yudu L3 | Jiangxi | CY-18 | *D. alata* | Honghe L2 | Yunnan |
| CY-34 | *D. alata* | Yudu L4 | Jiangxi | CY-90 | *D. alata* | Nuomi Yam | Yunnan |
| CY-35 | *D. alata* | Yudu L5 | Jiangxi |  |  |  |  |
| CY-36 | *D. alata* | Yudu L6 | Jiangxi |  |  |  |  |
| CY-72 | *D. alata* | Fulin L2 | Jiangxi |  |  |  |  |
| CY-196 | *D. alata* | Tianquan L1 | Sichuan |  |  |  |  |
| CY-208 | *D. alata* | Yashanyao 1 | Sichuan |  |  |  |  |
| CY-212 | *D. alata* | Miyi 2 | Sichuan |  |  |  |  |
| CY-235 | *D. alata* | Miyi W1 | Sichuan |  |  |  |  |
| CY-253 | *D. alata* | Miyizihua Yam | Sichuan |  |  |  |  |
| CY-246 | *D. alata* | Miyi 1 | Taiwan |  |  |  |  |
